# Availability, affordability, and associated factors of essential medicines in primary health care facilities of the Wolaita zone, southern Ethiopia: implication for access, a cross-sectional study, 2022

**DOI:** 10.1007/s43999-025-00078-w

**Published:** 2025-11-07

**Authors:** AtsedeTenna Adale

**Affiliations:** Department of Public Health, Wolaita Sodo University, Wolaita Sodo, Ethiopia

**Keywords:** Availability, Affordability, Essential medicines, Ethiopia

## Abstract

**Introduction:**

Access to essential medicines is fundamental to achieving universal health coverage. According to WHO, these medicines should be consistently available and affordable. However, in many low- and middle-income countries, health facilities experience frequent stockouts and high prices, limiting access and affordability. Such barriers reduce adherence, increase out-of-pocket spending, and compromise health outcomes. Assessing availability and affordability in primary health care facilities is therefore crucial to inform policy and promote equitable access.

**Objectives:**

To assess the availability and affordability of essential medicines and associated factors in Wolaita zone, Ethiopia, 2022.

**Methods:**

A facility-based cross-sectional study was conducted from February 1–30, 2022. Thirty public health facilities were selected, six from each of five districts. Sample size was determined using a single population proportion formula. Data were collected through patient exit interviews, entered into EpiData 3.1, and analyzed in SPSS version 25. Multivariable logistic regression was employed to identify factors associated with affordability.

**Results:**

Of 601 patients, 98% participated. The average availability of 26 selected core essential medicines was 56.53%. Among patients, 58.1% reported prescribed medicines as unaffordable. Factors significantly associated with affordability included level of health facility [AOR (95% CI) = 3.450 (2.275–5.231)], health status [AOR = 1.807 (1.027–3.179)], educational status [AOR = 3.413 (1.363–8.548)], and place of residence [AOR = 1.596 (1.019–2.551)].

**Conclusion:**

Essential medicine availability was low across facilities. Many patients were unable to obtain prescribed drugs due to unavailability and unaffordability. District and zonal health offices should ensure timely replenishment to strengthen access.

## Introduction

The Alma-Ata International Conference on Primary Health Care’s theme of “Health for All” in 1978 supports the case for equal access to life-saving medications and can be used as a benchmark to evaluate and improve national programs [1],. According to this strategy, the distribution of necessary medications and vaccinations is one of the foundational elements of primary health care delivery and serves as a key tool for healthcare professionals to help patients reach their health care objectives [2, 3].

The medications that meet the population’s top healthcare demands are considered to be essential. They are chosen with consideration for their relevance to public health, proof of their effectiveness and safety, and comparative cost-effectiveness [4]. A healthy healthcare system must have access to inexpensive, high-quality, and appropriate critical medications, vaccines, and technologies [5].

A crucial element of universal health care is free access to necessary medications. However, in low- and middle-income countries (LMICs), ensuring that everyone has access to vital medications is a significant difficulty. Although there is a fair amount of information about access to vital drugs from wealthy countries, it is frequently shaky and fragmented in LMICs. According to recent studies, only 44.3% of drugs are typically available in the public sector in LMICs [6].

The majority of the world’s top causes of death, including HIV/AIDS, infectious illnesses, and malaria, can only be adequately treated or prevented by having the right medications readily available at all times [7, 8]. The most common causes of discomfort, disability, and early mortality in low-income nations can be avoided, managed, or at the very least mitigated using affordable essential medicines [3]. Even so, hundreds of millions of people lack regular access to necessary medications. A third of the world’s population lacks access to necessary medications. The situation is worse in the most underdeveloped regions of Africa and Asia, where half the population cannot afford basic medications [9]. Many people who do have access to medicine are given the incorrect medication, receive insufficient treatment for their ailment, or misuse the medication. Patients are drawn when essential medications are accessible because they can then hear preventative and public health messages [3].

Essential medicine shortages are becoming more common worldwide, placing a strain on health systems. Reports of these shortages have come from high-, middle-, and low-income nations. They take up a lot of staff time and are expensive for health systems to manage, adding to the cost of replacing medications. Due to non-treatment, inadequate therapy, and potential medication errors from attempts to substitute missing medications, medicine shortages pose concerns for patient health [10].

Any healthcare system’s primary component is the supply of medications, and poor access to them jeopardizes the goals of equity, effectiveness, and health advancement. The world’s population lacks regular access to vital medications in about 30% of cases [9]. Health issues brought on by inadequate drug benefits are especially harmful in African nations, where it is estimated that 50–60% of the population lacks access to important medications [11].

The WHO states that 50% of all children under five who die worldwide from diseases like HIV, measles, pneumonia, diarrhea, and malaria do so in Africa [12].

Ethiopia is impacted by the lack of funding for healthcare, which results in regular drug shortages in public health institutions. Therefore, the fee waiver scheme did not protect patients from having to pay for their medications. Furthermore, only 0.2% of all drug expenditures in 2005–2006 were covered by employer-provided insurance [13]. Patients are forced to forgo therapy altogether or switch to the more expensive private sector when there are no medications available in the public sector, which exposes them to out-of-pocket (OOP) costs. Therefore, medications can account for more than half of the real cost of a visit, raising the possibility of experiencing catastrophic medical costs and the related dangers of being impoverished. Health facilities in the public sector play a crucial role in ensuring that the poor have access to medicines because they typically offer these services for free or at a reduced cost [14, 15].

The impact on private OOP expenses is greatly lessened by ensuring free access to necessary medications. Additionally, it offers the population’s most vulnerable members financial risk protection against catastrophic medical expenses [16]. There is little or no evidence about the availability and affordability of essential medicine in Wolaita area, which is one of the densely populated areas in southern Ethiopia, despite efforts to improve primary health care delivery, ensuring continuous access to essential medicines remains a major challenge in the area.

However, there is insufficient empirical evidence that comprehensively assesses these issues within the zone. where access to essential medicines remains a public health concern. therefore, the purpose of this research is to assess the availability, affordability, and associated factors influencing the access to essential medicines in Wolaita zone. It also aims to understand the key barriers to accessing essential medicines and to assess the implications these factors have on public health and healthcare equity.

## Methods

### Study design and setting

An institution-based cross-sectional study was conducted in Wolaita zone, from February 1 to 30, 2022. Wolaita zone is one of the 16 zones in South Nations Nationalities and Peoples Region (SNNPR), and about 330 KM distance from capital city, Addis Ababa. The zone is divided in to 16 districts and 6 city administration with a total projected population of 2,114,382. In Wolaita zone, there are 1 referral hospital (Wolaita Sodo University Specialized Teaching Hospital, 7 primary hospitals, 67 health centers, and 384 health posts.

### Study participants

All sampled Patients who visited the selected primary health care facilities with medicine outlets in the Wolaita Zone during the study period were included. Patients with fee waivers and patients enrolled with community-based health insurance (CBHI) were excluded from the sample.

### Sample size and sampling techniques

The sample size for health facilities was established in accordance with the WHOs operational package for measuring, monitoring, and evaluating the country’s pharmaceutical situations [17]. Based on this approach, six public health facilities were picked from each of the five designated districts for a total of 30 public health facilities chosen. A single population proportion formula was used to calculate sample size of patients as given in the equation;

$${\rm{n = }}{\left( {{\rm Z}{{\rm{ }}_{1 - \alpha /2}}} \right)^2}{\rm P}{\rm{ }}\left( {1 - {\rm P}} \right)/{{\rm{d}}^2}$$

The assumptions: level of confidence 95%, 5% margin of error, and proportion of affordability of essential medicines in Jimma zone is 37.1% [18].

$${\rm{n = }}{{\rm Z}^2}{\alpha _{/2}}{\rm{pq/}}{d^2} = {1.96^2}x0.371x0.639/{0.05^2} = 364$$

Where, *n* = sample size, Zα/2 = Critical value = 1.96, *p* = proportion of Affordability of essential medicines in Jimma zone = 37.1% d = precision (margin of error) = 0.05. Using a design effect of 1.5 for multistage sampling and 10% of non-response rate, the final sample size was *n* = 601

The sample process followed the WHOs advice. It suggests choosing five locales or geographic regions for evaluating drugs in healthcare facilities. To choose these five districts, one should be the capital city or town, one should be the most rural or remote district, and three other geographical areas or districts should be chosen at random from the other districts [17]. With the exception of six health facilities being picked inside each district, the same guideline suggests choosing 30 public health facilities. In accordance with this theory, one health facility was chosen as the primary (largest) public facility or hospital in the region, the other facility as the lowest level public health center or the most rural health center, and the final four facilities were chosen at random from all of the remaining primary and other public health facilities within each district. As a result, 30 public health facilities from five districts, comprising six facilities from each district, were chosen. For the exit interview, the total sample size was proportionally distributed for each of the chosen healthcare institutions based on the average daily patient volume for the outpatient department as reported in the HMIS report for the prior year’s comparable month. Lastly, patients were questioned.

### Data collection

On the day of data collection, availability of critical medicines was assessed by reading stock cards, browsing the shelves, and recognizing the stated key medications. According to the World Health Organization/Health Action International (WHO/HAI) procedures, drug prices were entered on a data compilation sheet [19]. The branch of the Pharmaceutical Fund and Supply Agency, which distributes medications to public health facilities, is located in the middle of the study area and is where the information for public procurement pricing was gathered. Data regarding availability was confirmed by direct observation. IPR is included in these lists of medications in the dosage and potency that are indicated.

These lists of medicines were taken from the 2014 national essential medicines list of Ethiopia and the national standard treatment guideline to treat the most common health problems in the study area. Medicines were selected based on the top fifteen morbidities of the study area that can be treated as outpatients from the health facilities. The most recent price data was obtained from stock records and asking a person in charge of pharmacy in medicine outlets and storekeepers for medicines that have no stock records.

Patient exit interview data was collected by using pretested interviewer-administered structured questionnaires to assess affordability and its associated factors. The questionnaires are prepared by reviewing different literatures of similar studies and modified according to local context. It was developed in English and translated into local languages, ‘wolaitegna’ and ‘Amharic,’ by language experts. The tools were pretested at 10% of the sampled facilities prior to its actual use and improved in terms of their clarity, understandability and simplicity. Two-day training was given for data collectors and supervisors

## Measurements and scoring

**Availability:** Refers to the physical accessibility and presence of any quantity of the medicines with the specified dosage form and strength that are in stock on the day of the survey in the primary health care facilities of Wolaita Zone [17, 19, 20].

% of each key essential medicine available = the sum of each key essential medicine surveyed in all study facilities * 100/the number of health facilities sampled.

Average availability = the sum of percentages of each key essential medicine available/number of lists of key essential medicines prepared as checklists.

**Classification of Availability** [21]

**Very low**: If it is less than 30%

**Low**: If it is 30–49%

**Fairly high**: If it is 50–80%

**High**: If it is > 80%

**Perceived Affordability** is a result of clients’ perception of the Medicine that costs the equivalent to (less than or equal to) one day’s salary of the lowest-paid government worker to purchase for diagnosed diseases. It is classified as affordable if the lowest-paid government worker were able to obtain the prescribed medicines and not affordable if they were not able to obtain.

**Affordability Based on Selected Diseases:** Medicine that costs the equivalent to (less than or equal to) one day’s salary of the lowest-paid government worker to purchase a full course of treatment for an acute condition or a one-month course of treatment for a chronic condition was generally considered affordable; treatment that costs more than this was considered unaffordable [19]. At the time of the survey, the lowest-paid unskilled government worker earned 1100 ETB per month (US$ 0.73) per day with a currency exchange rate of 1 US$ = 50.6 ETB.

**Days Wages** = median medicine price found in the facilities times the number of units in a standard course of treatment/daily salary of the lowest paid unskilled government worker [17].

## Data analysis

Facility-based data on the availability and prices of medicines were double-entered into the World Health Organization/Health Action International (WHO/HAI) availability and price Workbook software, which has an auto-analysis feature.

The accuracy and completeness of quantitative data were verified before it was modified, coded, entered into Epi-data 3.1, and exported to SPSS version 25 for cleaning and analysis. In order to display the quantitative results, descriptive statistics such as frequency, mean, percentile, median, and standard deviation were generated. The data were then presented in tables, graphs, and numerical summaries. Using bivariate analysis, it was possible to pinpoint the variables influencing the cost of necessary medications. Multiple logistic regressions were considered possibilities for variables with p-values of 0.25 in bivariate analysis. [22]

The backward stepwise method was used to do several logistic regressions in order to find factors that were independently linked to the dependent variables. Using the odds ratio and 95% confidence intervals, the strength of the link was calculated. Statistical significance was defined as a P-value of 0.05 or lower.

Ethical clearance was initially obtained from Institutional Review Board (IRB) of College of health sciences and medicine, Wolaita Sodo University. Formal letter of cooperation was written to Wolaita Zone Health Department and respective facilities. After clear discussion about the actual study or explaining of purpose of the study, verbal informed consent was obtained from each study subjects. Identification of study participants by name were avoided to assure the confidentiality of the information obtained.

## Results

### Socio-demographic and socioeconomic characteristics of respondents

In this study, out of 601 patients sampled, 590 responded to the questionnaires, making a response rate of 98%. Out of the patients, 302 (51.2%) were females and 361 (61.2%) were married, while only 24 (4.1%) were divorced. Regarding the residential area, 326 (55.3%) were urban dwellers. Moreover, 145 (24.6%) of them had two family members being economically dependent (Table 1).

**Table 1:** Socio-demographic and socioeconomic characteristics of patients

	Socio-demographic factors	Category of characteristics	Frequency (%)
1	Age	18–29	230 (39%)
		30–44	214 (36.3%)
		45–64	135 (22.9%)
		≥65	11 (1.9%)
2	Sex		
		Male	288(48.8)
		Female	302(51.2)
3	Residence		
		Urban	326(55.3)
		Rural	264(44.7)
4	Occupation		
		Farmer	129(21.9)
		Merchant	115(19.5)
		Student	131(22.2)
		House wife	86(14.6)
		Government employee 118(20)	
		Daily laborer	6(1%)
		Others*	5(0.8)
5	Marital status		
		Single	196(33.2)
		Married	361(61.2)
		Divorced	24(4.1)
		Widowed	9(1.5)
6	Monthly income		
		≤10$ ≤520 ETB	263(44.6%)
		(10–50) $(520–2600 ETB) 172(29.2%)	
		(50–115) $ (2600-5980ETB) 94(15.9%)	
		(115–170) $(5980–8840)	61(10.3%)
7	Educational status		
		Illiterate	93(15.8)
		Grade 1–8	196(33.2)
		Grade 9–12	167(28.3)
		Collage and above	134(22.7)
8	Number of economically dependent family member		
		None	182 (30.8)
		One	127 (21.5)
		Two	145 (24.6)
		Three	77 (13.1)
		Four	50 (8.5)
9		More than four	9 (1.5)
	Service sought for		
		Self	519(88%)
		Free	389(65.9%)
		Paying	
10	Self-reported health status	Good and above	295(50%)
		Fair or poor	295(50%)

### Health-facility- and health-service-related characteristics

Three hundred ninety-five (66.9%) of the total patients who visited the assessed healthcare facilities came from clinics, while 195 (33.1%) came from hospitals. In terms of the average number of medications given to each patient, there were 2.42. medications. 320 patients (54.2%) had less than two delivered medications per prescription, 216 (28.1%) received two or more medications per prescription, and 104 (17.6%) received no medications at all.

### Availability of essential medicine

#### Availability of selected core essential medicines

The average availability of generic medications was 56.53%, based on 26 core stock lists of critical medications. In hospitals and health facilities, the average availability was discovered to be 51.69% and 63.52%, respectively (Table 2). During the study period, none of the examined health institutions had all 26 of the key lists of medications. The most frequently unavailable medications among the essential ones surveyed were omeprazole 20 mg (average availability of 12.5%), glibenclamide 5 mg and salbutamol 0.1 mg/dose, aerosol/puff (average availability of 25% each), and ceftriaxone 1 gm IV (average availability of 37.5%). (Table 2). In terms of the effectiveness of individual medications, 2 (7.69%), 20 (76.9%), 1 (3.8%), and 3 (11.5%) lists of medications were rated as high ( > 80%), fairly high (50–80%), low (39–49%), and extremely low (30%), respectively.

**Table 2:** Average availability of individual essential medicines by level of facility

S.N	Names of essential medicines, strength and dosage (N = 30)	Average availability in (%)	Average availability in (%)	Average availability by level of facility (%)
			**Hospitals(N = 4)**	**HCs(N = 26)**
1	Amoxicillin 500 mg cap	75	66.6	80
2	Amoxicillin +clavulanic acid	50	33.6	40
3	Ciprofloxacin 500 mg tab	62.5	75	80
4	ORS sachet	87.5	50	75
5	Doxycycline 100 mg cap/tab	87.5	66.6	80
6	Metronidazole 250 mg cap	75	66.6	80
7	Sulphamethoxazole+trimethoprime	50	33.3	60
8	Mebendazole 100 mg tab	62.5	100	40
9	Diclophenac 75 mg/3 ml	62.5	66.6	80
10	Cotrimoxazole 240 mg/5 ml	50	33.3	60
11	Amoxicillin 125 mg/5 ml	62.5	16.6	80
12	Paracetamol 500 mg tab	62.5	66.6	80
13	Chloroquine 250 mg	50	33.3	60
14	Norfloxacilline 400 mg tab	62.5	33.3	80
15	Omeprazole 20 mg	12.5	33.3	0
16	Praziquantel 600 mg tab	62.5	33.3	80
17	Ceftriaxone 1 gm IV	37.5	33.3	40
18	Ringer lactate solution	50	66.6	60
19	Cloxacillin 500 mg, tab	62.5	66.6	66
20	Salbutamol 0.1 mg/dose, aerosol/puff	25	50	-
21	Gentamycin	60	61.5	50
22	Primaquine phosphate	53.3	30.8	50
23	Artemether lumefantrin (20/120 mg)	62.5	42.3	80
24	Glibenclamide 5 mg tab	25	66.6	-
25	Prednisolone	62.5	66.6	60
26	40% glucose	60	50	66.6
	Average percent availability	**56.53**	**51.69**	**63.52**

### Affordability of essential medicines based on selected diseases

With basic treatment costing a day’s wage or less of the LPGW, the affordability of generic medications at the studied healthcare facilities varied for the majority of diseases. For ten conventional treatments, the minimum daily salary was an average of 1.74 (with a range of 0.02 to 4.3). Typhoid fever, adult pneumonia, urinary tract infection (UTI), skin and subcutaneous tissue infection, typhus, and diabetes mellitus were among the illnesses for which LPGW treatments cost more than one day’s pay. Peptic ulcer disease (PUD), helminthiasis, giardiasis, and arthritis were among the LPGW conditions for which treatment was less expensive than one day’s pay. In order to buy a course of diclofenac tablets and omeprazole capsules to treat peptic ulcer disease and arthritis, respectively, an LPGW would need to work on average for 0.4 days. In the health facilities surveyed, the LPGW would need to labor 3.1 days to afford the LPG Ceftriaxone injection for the treatment of typhoid fever. (Table 3).

**Table 3:** Affordability of a single course of treatment in days’ wage of an LPGW for some disease conditions

**Disease condition**	**Drug name, strength, and dosage forms**		**Standard Rx schedule**	**Affordability in days of wages (ETB) Ethiopian birr**	**Affordability in days of wages ($) US dollar**
Typhoid fever	Ciprofloxacillin 500 mg tab		1-tab x2/day x7 = 14 tabs	50	0.99
	Ceftriaxone 1 gm Iv, vial		1 vial x1/day ×7 = 7 vials	217	4.3
Adult pneumonia	Amoxicillin 500 mg tab		1 tab ×3/day ×7 day	60	1.19
	Amoxicillin acid	clavulanic	1 tab ×3/day ×7 day	127	2.5
PUD	Omeprazole 20 mg cap		1 cap x1/day for 30 days = 30 caps	30	0.6
Helminthiasis	Albendazole 400 mg tab		1 tab stat	1	0.02
	Albendazole susp.	100 mg/5 ml	5 ml stat	24	0.48
UTI	Norfloxacillin 400 mg tab		1-tab x2/day x7 = 14 tabs	56	1.1
Skin and Subcutaneous tissue infection	Colloxacillin 500 mg tab		1 tab ×4/day ×14 day = 14 tabs	52	1.07
Typhus	Doxycycline 100 mg, cap		1 tab ×2/day for 7 days = 14 tabs	47	0.93
Giardiasis	Metronidazole 500 mg cap		1 caps ×3/day ×7 days = 42 caps	29	0.57
Arthritis	Diclofenac 50 mg tab		1 tab ×2/day for 30 days = 42 tabs	28	0.55
Diabetic mellitus	Glibenclamide 5 mg cap		1 tab ×2/day for 30 days = 60 tabs	108	2.13

### Affordability of dispensed medicines in days wages of an LPGW

**Fig. 1:**
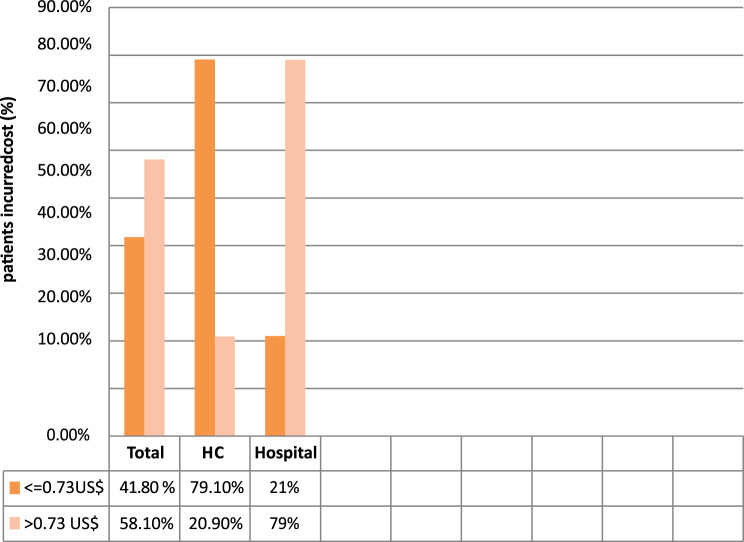
Percentage of patients who incurred costs on medicines in relation to the daily wage of an LPGW (0.73 usd). Were HC : health center

Patients who visited the surveyed health institutions reported spending, on average, US$ 1.24 (1.7 days’ wage) on medications, with US$ 1.09 (1.5 days’ wage) being the median expenditure. 343 patients (58.1%) who received prescription drugs paid more than $0.73 (the lowest daily government employee wage) in expenditures. According to health institution categories, 271 (79%) and 72 (20.99%) patients in hospitals and health centers, respectively, spent more than US$ 0.73 on prescription drugs (Fig. 1).

### Affordability of essential medicines based on consumers’ perception

Out of all patients, 422 (71.5%) did not receive any medication from a public health facility, whereas 137 (23.2%) and 31 (5.3%) totally and partially purchased medications from public health stores and institutions, respectively. 408 (69.2%) of the patients who did not or only partially obtained medications proceeded to purchase the remaining medications from the private sector, while 78 (13.2%) and 101 (17.6%) chose traditional medicines and received only a partial course of therapy at home. The biggest barriers to obtaining the necessary drugs from the assessed health institutions were their unavailability and cost. 247 (41.9%) patients felt that the provided medications were reasonably priced.

### Factors associated with perceived affordability of essential medicines

Nine of the 14 variables in the bivariate analysis had a p-value under 0.25, making them eligible for the multivariable logistic regression analysis. The degree of the health facility, educational level, quantity of medications dispersed, health status, location, sex, age, income, and the number of family members who are economically dependent were all candidates for multivariate analysis, according to bivariate analysis.(Table 4).

**Table 4:** Bivariate analysis of factors associated with affordability of essential medicines

Variable name	Category	Not affordable N (%)	Affordable N (%)	*p* value	95% CI for COR
Age	18–29	125 (34.4%)	105 (42.5%)	0.288	0.560,1.188
	30–44	127 (37%)	87 (35.2%)	0.184**	0.484,1.150
	45–64	83 (24.2%)	52 (21%)	0.242**	0.115,1.726
	≥65	8 (2.3%)	3 (1.2%)		1
Sex	Male	179 (52.2%)	109 (44.1%)	0.054*	0.52,1.005
	Female	164 (47.8%)	138 (55.9%)		1
Place of residence	Urban	178(51.9%)	148 (59.9%)		1
	Rural	165 (48.1%)	99 (40.1%)	0.053*	0.995,1.930
Present health status	Good	163 (47.5%)	132 (53.4%)		1
	Fair	106 (30.9%)	80 (32.4%)	0.023	1.078,2.720
	Poor	74 (21.6%)	35 (14.2%)	0.065	0.972,2.625
Estimated monthly income					
	≤1100ETB	180 (52.5%)	142 (57.5%)	0.228**	0.881,1.703
	> 1100ETB	163 (47.5%)	105 (42.5%)	1	
Educational status	Illiterate	32 (9.3%)	61 (24.7%)	0.002**	1.361,4.062
	1–8	136 (39.7%)	60 (24.3%)	0.009**	0.345,0.859
	9–12	101 (29.4%)	66 (26.7%)	0.329	0.508,1.277
	Collage &above	74 (21.6%)	60 (24.3%)		1
Place of visit	Health center	72 (21%)	123 (49.8%)	0.000**	2.604,5.352
	Hospital	271 (79%)	124 (50.2%)		1
Number of dispensed medicines	≤2	134(39.1%)	74 (30%)		1
	> 2	146 (42.6%)	141 (57.1%)	0.749	0.652,1.813
	None	63 (18.4%)	32 (13%)	0.009**	1.171,3.086
Economically dependent family members	≤ one	98 (28.6%)	84 (34%)	1	
	> one	245 (71.4%)	163 (66%)	0.159**	0.906,1.833

** Candidate variables with *p* < 0.25.

In order to find variables that were independently related with an outcome variable, the affordability of necessary medications, those variables with a p-value of less than 0.25 were once more put into multiple logistic regression models. In a multivariable logistic regression study, factors having a p-value of less than 0.05 were considered significant predictors of the outcome variable. The final model demonstrated a statistically significant relationship between health facility categories and the cost-effectiveness of dispensed medications (p-value 0.001). Patients who visited health centers were 3.450 times more likely to receive the dispensed medications at affordable prices than patients who visited hospitals [OR (95% CI) = 3.450 (2.275, 5.231)]. In this study, urban residents were 1.596 times more likely to be able to get the prescribed medications at reasonable prices than rural residents [OR (95% CI) = 1.596 (1.019, 2.551)].

The study also revealed that participants with a secondary level of education were 3.4 times more likely to obtain prescribed medications at reasonable prices compared to those with lower educational attainment. Furthermore, patients who reported having fair health status were 1.8 times more likely to obtain their prescribed medications compared to those who reported poor health status. (Table 5).

**Table 5:** Multivariable logistic regression analysis of factors associated with affordability of essential medicine

Variables name	Categories	Not affordable	Affordable	P value	AOR (95% CI)
Level of health facility	Health center	72 (21%)	123 (49.8%)	0.000**	3.450 (2.275,5.231)
	Hospital	271 (79%)	124 (50.2%)		1
Present health status	Good	163 (47.5%)	132 (53.4%)		1
	Fair	106 (30.9%)	80 (32.4%)	0.040**	1.807 (1.027,3.179)
	Poor	74 (21.6%)	35 (14.2%)	0.459	1.242 (0.700,2.206)
Place of residence	Urban	178(51.9%)	148 (59.9%)	0.040**	1.596 (1.019,2.551)
	Rural	165 (48.1%)	99 (40.1%)		1
Educational status	Illiterate	32 (9.3%)	61 (24.7%)	0.255	0.691 (0.365,1.306)
	1–8	136 (39.7%)	60 (24.3%)	0.218	0.648 (0.325,1.292)
	9–12	101 (29.4%)	66 (26.7%)	0.009**	3.413 (1.363,8.548)
	Collage and above	74 (21.6%)	60 (24.3%)		1

AOR Adjusted odds ratio, CI Confidence interval, 1 Reference group. *Statistically significant at *p* < 0.05

## Discussion

One of the most crucial goals of any national drug policy is to increase access to essential medications. However, for the majority of people, particularly the poor who depend on these medications, their lack of accessibility and high cost remain major issues [7]. Medicines must be completely accessible in the public health facilities sectors for patients to have enough access to necessary medications [3]. This study shows that 56.53% of chosen public health facilities have an average availability of critical medications (*n* = 26). This result is less than that of a study done in the Amhara region of Ethiopia, the Jimma region of Uganda, and the Kaliro district of Uganda, which revealed average availability of 79.5%, 78.6, and 91% at the location of data collection in public health institutions [18, 23, 24]. The variation may be caused by the limited number of various types of critical medications chosen in the case of the prior study and the varying incidence of medical conditions in the two study areas.

The results of this study are, however, a little higher than those of a study done in Jimma Health Center seven years ago, which found that an average of 55.65% of critical medications were available at the time of data collection [21]. The findings may have varied due to the lengthy time gap between the two research projects and seasonal variations, which affect the quantity of medication intake in the examined healthcare institutions.

When determining the cost-effectiveness of a single course of therapy for particular disorders, it is assumed that the average daily wage of the lowest-paid unskilled government employee is similar to the LPGW salary per individual level [19]. The findings revealed that the costs of treating typhoid fever, adult pneumonia, urinary tract infection (UTI), skin and subcutaneous tissue infection, typhus, and diabetes mellitus were higher than those suggested by a study in Jimma [18]. The variation may be a result of a prior study’s data collection period’s lower median price for medications at healthcare facilities. The lack of a defined medicine pricing policy, significant retail markups, wide price variance, and the absence of a system of pharmaceutical review all contributed to higher drug prices in Ethiopian healthcare institutions [25].

Most of the data in this study indicated that the chosen medications were not within the budgets of the assessed healthcare facilities for treating common ailments. This is due to the fact that only one medication was considered, and the affordability estimates assumed a high wage relative to what was reported in other studies.

Although affordability was only considered in terms of one medication, most illnesses are really treated with a combination of medications, so this measurement may not be enough. This study, which discovered that the typical number of medications per prescription was 2.42, supported this conclusion.

Patients in this study paid an average of US$ 1.24 (1.7 days’ wages) out-of-pocket for medication, which is low compared to the cost reported by a study carried out in Jimma, Ethiopia which was US$ 3.22 (3.07 days’ wage) [18].

The average number of medications per prescription in this study was 1.7, which was similar to the 1.72 in a prior study. Given this amount and the LPGWs wage, a patient would need to labor more than one day to pay for their prescription medications. The exorbitant price of medications is a direct cause of the catastrophic health expenditure and the resulting risk of poverty [15].

In this study, 58.1% of the participants described the prescription drugs as being out of their price range. In comparison to the study’s results from the Jimma Health Centre, which were reported at 47.8% [21], this figure is greater. The mean variance may result from the classification of the outcome variable at a different level in the two studies. In a prior study, the outcome variable had three levels; in this study, it had only two levels.

Due to the expensive expense of prescribed medications, patients may choose to forego them. However, doing so can result in major health issues and unneeded medical difficulties [26].

The results of this study showed that patients from health centers were more likely than patients from hospitals to receive prescribed medications at reasonable costs. An investigation was carried out in a different part of Ethiopia in 2015 [20] that lends weight to this conclusion. This is supported by the study’s findings. Compared to health facilities, hospitals often prescribe drugs for chronic conditions for longer durations, which may be more expensive than drugs for acute conditions.

Another significant predictor of perceived affordability of necessary medications was educational attainment. According to this study, patients who had completed high school or above were more likely than illiterate or primary school students to be able to pay for the prescribed medications. This result is consistent with research from Nigeria, which found that a person’s level of education affects their capacity to pay for prescribed medications [26]. The health status at the time of the visit was also discovered to be a significant predictor of the perceived affordability of medicines prescribed in this study. Compared to individuals with low health status, patients with a fair health status were more likely to find cheap medications. According to the study’s findings, patients from metropolitan areas had a higher likelihood of finding affordable medications than those from rural locations. This can be the case because urban residents have more sources of income than rural residents do. This was supported by the study’s findings, which showed that persons from urban regions had a mean income of US$25.2, which was higher than that of people from rural areas, which was US$18.5.

## Conclusions and recommendations

According to the availability classification, the average availability of critical medications was rather high. However, the availability of essential, inexpensive critical medications and cost-free anti-malarial medications was low in the examined health institutions. In this study, a large number of patients seeking treatment in public health facilities fail to acquire a sizable share of the prescribed medications due to their unavailability and unaffordability.

In the examined healthcare institutions, more than half of the core basic medications used to address common health issues were unaffordable. 58.1% of the patients who visited the surveyed healthcare facilities during the study period could not pay the cost of the medications that were prescribed to them. The perceived affordability of important medications was positively correlated with the quality of the medical facility, educational level, residential status, and health state at the time of the visit.

Even if there are community-based health insurance schemes, district health offices and zonal health departments should increase access to critical medications as soon as they go out of stock, especially free medications.

The health insurance schemes should be implemented effectively, and healthcare professionals should improve their prescription patterns to reduce the amount of money patients waste on unnecessary medication costs.

## Data Availability

The datasets used and analyzed during the current study are available from the corresponding author on reasonable request.
