# Correction: Availability, affordability, and associated factors of essential medicines in primary health care facilities of the Wolaita zone, southern Ethiopia: implication for access, a cross-sectional study, 2022

**DOI:** 10.1007/s43999-026-00087-3

**Published:** 2026-02-03

**Authors:** AtsedeTenna Adale

**Affiliations:** Department of Public Health, Wolaita Sodo University, Wolaita Sodo, Ethiopia

**Correction: Res Health Serv Reg (2025) 4:18**

10.1007/s43999-025-00078-w

Following publication of the original article [1], the authors would like to correct Wolaita region to Wolaita zone in the article title, Abstract and in the final paragraph of the Introduction.

The original article [1] has been corrected.
